# Natural Metabolites as Modulators of Sensing and Signaling Mechanisms: Unlocking Anti-Ovarian Cancer Potential

**DOI:** 10.3390/biomedicines13081830

**Published:** 2025-07-26

**Authors:** Megha Verma, Prem Shankar Mishra, SK. Abdul Rahaman, Tanya Gupta, Abid Ali Sheikh, Ashok Kumar Sah, Velilyaeva Aliya Sabrievna, Karomatov Inomdzhon Dzhuraevich, Anass M. Abbas, Manar G. Shalabi, Muhayyoxon Khamdamova, Baymuradov Ravshan Radjabovich, Feruza Rakhmatbayevna Karimova, Ranjay Kumar Choudhary, Said Al Ghenaimi

**Affiliations:** 1 School of Medical & Allied Sciences, Galgotias University, Greater Noida 201310, Uttar Pradesh, India; vmegha321@gmail.com (M.V.); rahamandr123@gmail.com (S.A.R.); tanyagupta@galgotiasuniversity.edu.in (T.G.); 2 Faculty of Pharmacy, Vidya University, Meerut 250002, Uttar Pradesh, India; 3 National Institute of Biologicals, Noida 201309, Uttar Pradesh, India; abidalis2007@gmail.com; 4 Department of Medical Laboratory Sciences, College of Applied and Health Sciences, A’ Sharqiyah University, Ibra 400, Oman; 5 Department of Psychiatry, Medical Psychology, and Narcology, Samarkand State Medical University, Samarkand 140104, Uzbekistan; alsabvel2@gmail.com; 6 Department of Folk Medicine and Professional Diseases, Bukhara State Medical Institute, Bukhara 200101, Uzbekistan; inom65@rambler.ru; 7 Department of Clinical Laboratory Sciences, College of Applied Medical Sciences, Jouf University, Sakaka 72341, Saudi Arabia; anasseen@hotmail.com (A.M.A.); dr.mpathology@gmail.com (M.G.S.); 8 Bukhara State Medical Institute (Abu Ali Ibn Sina), Bukhara 200118, Uzbekistan; hamdamova.muhayyoxon@bsmi.uz; 9 Department of Anatomy, Clinical Anatomy (OSTA), Bukhara State Medical Institute, Bukhara 140100, Uzbekistan; baymuradovravshan@gmail.com; 10 Department of Traditional Medicine, Occupational Diseases and Allergology, Abu Ali ibn Sino Bukhara State Medical Institute, Bukhara 140100, Uzbekistan; feruzakfr77@gmail.com; 11 Department of Medical Laboratory Technology, UIAHS, Chandigarh University, Mohali 140413, Punjab, India; r.choudharymt@gmail.com; 12 School of Allied and Health Sciences, Sanskaram University, Jhajjar 124108, Haryana, India

**Keywords:** natural metabolites (alkaloids, flavonoids, and terpenoids), ovarian cancer, molecular mechanism, signaling pathways

## Abstract

Cancer presents significant challenges owing to its complex molecular pathways and resistance to therapy. Natural metabolites have significant medicinal potential by regulating the sensing and signaling pathways associated with cancer development. Recognizing their interactions within the tumor microenvironment may unveil innovative techniques for inhibiting malignant activities and improve therapy success. This article highlights studies regarding ovarian cancer metabolism, signaling mechanisms, and therapeutic natural substances. This study summarizes clinical and experimental results to emphasise the synergistic effects of alkaloids, flavonoids, and terpenoids in improving therapeutic effectiveness and alleviating drug resistance. Bioactive compounds are essential in regulating ovarian cancer metabolism and signaling pathways, affecting glycolysis, lipid metabolism, and the survival of tumor cells. This review examines metabolic programming and essential pathways, including glycolysis, TCA cycle, lipid metabolism, PI3K/AKT/mTOR, AMPK, and MAPK, emphasizing their therapeutic significance. The integration of metabolic treatments with medicines based on natural compounds has significant potential for enhancing treatment effectiveness and mitigating therapeutic resistance. Ovarian cancer needs an integrated strategy that includes metabolic reprogramming, signaling modulation, and drugs derived from natural products. Natural chemicals provide intriguing approaches to address chemotherapy resistance and improve treatment efficacy. Further research is required to enhance these methodologies and evaluate their practical applicability for improved patient outcomes.

## 1. Introduction

Cancer is a severe issue that impacts every human society’s health. Regretfully, the illness exhibits variability at the tissue level, which poses significant challenges for both precise diagnosis and therapy success [1,2]. The prostate, lung and bronchus, colon or rectum, and urinary bladder have the largest percentages for cancer types in males, correspondingly. The breast, lung and bronchus, colon or rectum, vaginal corpus, and thyroid are the areas in women where cancer incidence is highest [3]. The malignancies that affect children most frequently are blood cancer, brain cancer, and lymph node cancer, in that order [4,5]. A sequence of progressively more severe gene mutations that alter cell activities leads to cancer. Chemical substances have a role in the formation of cancer cells and gene alterations. Moreover, smoking contains a number of chemical components that cause cancer and lung cancer [6]. Fascinatingly, chemicals found in the environment have the potential to cause cancer, affecting cells’ cytoplasm and nucleus, either directly or indirectly, resulting in genetic abnormalities and gene mutations [7,8,9,10].

One of the worst gynecologic malignancies, ovarian cancer, is expected to kill 13,940 people in 2020 (American Cancer Society), making up 5% of all cancer fatalities in women [11,12]. At baseline in 2022, four in five women in Ireland were not confident in recognizing OCa symptoms. Despite two highly successful awareness campaigns reaching audiences of >8 million, similar percentages are not confident to recognize symptoms in 2024 [13]. Less than 30% of patients with ovarian cancer survive for five years after receiving a diagnosis in the advanced stages (III and IV) in more than 70% of cases [14,15]. The primary causes of these bad clinical outcomes are delayed diagnosis and chemotherapy-resistant malignancy, which promotes disease development and extremely aggressive metastases [16]. Therefore, it is essential to find novel biomarkers for early detection and new preventive techniques to improve clinical results. Due to their involvement in numerous cancer hallmarks, lengthy non-coding RNAs (lncRNAs), or non-coding RNA transcripts longer than 200 nucleotides. It has been shown in numerous studies to represent potential therapeutic targets and diagnostic biomarkers [17,18].

The pathophysiology of OCa has been definitively linked to hormonal and reproductive variables by epidemiological studies. To suit the evidence, two main theories have arisen [19]. According to the “incessant ovulation” theory, the frequency of ovulatory cycles raises the rate of cellular division linked to surface epithelium repair following each ovulation, which in turn increases the frequency of spontaneous mutations. The relationship between an increased lifetime ovulation counts and an increased risk [20,21,22,23].

### 1.1. Sensing and Signaling Pathway in Ovarian Cancer

Cellular metabolism fuels the growth, unchecked multiplication, invasiveness, and metastasis of cancer cells. In contrast to their normal counterparts, a number of metabolic pathways were changed in cancer cells to help them endure and adapt to the shifting conditions within the tumor microenvironment. Even though Hanahan and Weinberg identify altered metabolism as a significant cancer characteristic, it is one of the least researched [24]. The various ovarian cancer pathways are as follows.

#### 1.1.1. Glycolytic Pathways

**Figure 1:**
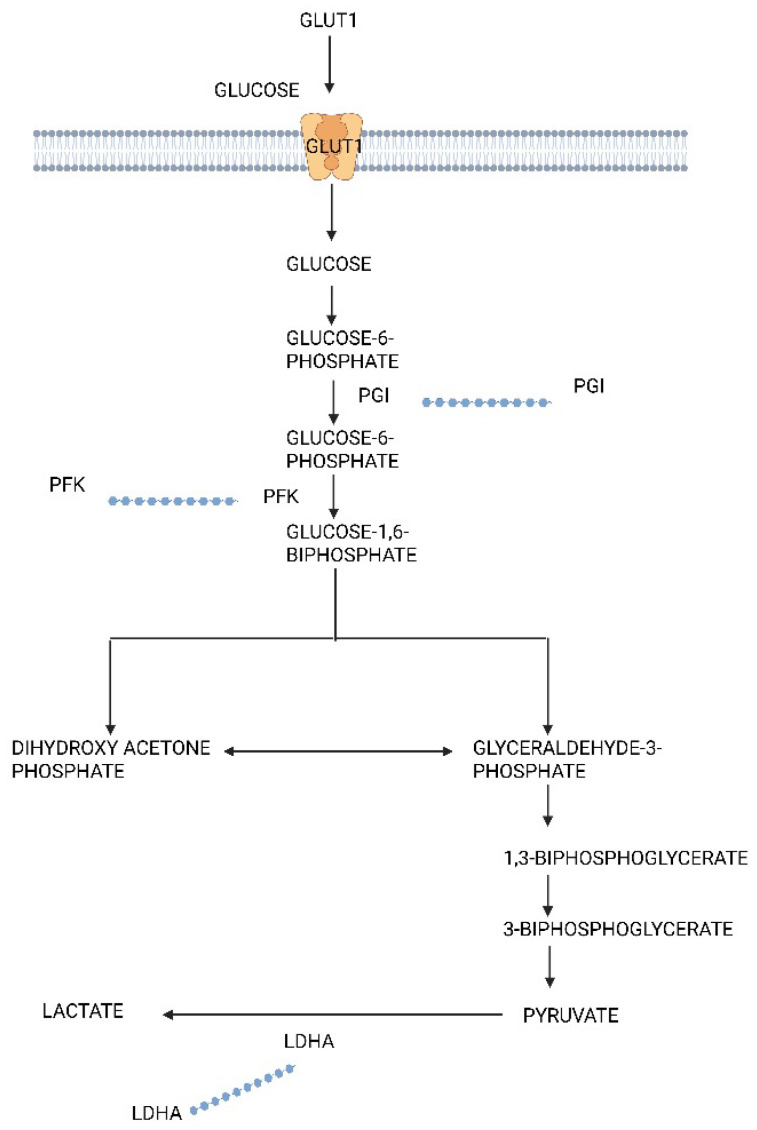
Differentially expressed proteins of the glycolysis pathway in ovarian cancer and highlight of key differentially regulated genes (GLUT1; overexpressed, PFK; underexpressed, PGI; overexpressed, LDHA; overexpressed).

Glycolysis is a metabolic process that separates glucose into two molecules with three carbons each. It occurs in the cell cytoplasm under anaerobic conditions. Cancer cells undergo a modified form of glycolysis called aerobic glycolysis or the Warburg effect in which the cells rapidly proliferate, and there is an increased glucose uptake and lactate production even in the presence of oxygen [25]. Figure 1 displays the glycolysis pathways’ differentially expressed proteins as boxplots downloaded from the GEPIA web server by [26]. When aerobic conditions are present, tumor cells use glycolysis to produce over 60% of the ATP [27]. The Warburg effect is associated with the development of epithelial ovarian cancer (EOC). Teng et al. revealed that the inhibition of AKT2 and AKT3 serine/threonine kinases, which activate downstream of the PI3K signaling pathway, significantly alters this metabolic transition, thereby affecting tumor progression [28].

A transcription factor called hepatocyte nuclear factor one (HNF1) plays a role in the formation of kidney and pancreatic beta cells. The promotion of increased glucose absorption and increased aerobic glycolysis in OCCC was linked to altered glucose metabolism through overexpression of (hepatocyte nuclear factor one) HNF1 [29]. Nitric oxide (NO) metabolic reprogramming is linked to tumor growth in cancer, particularly in ovarian cancer. According to Caneba et al., NO plays a role in controlling tumor growth and suppressing mitochondrial respiration in ovarian cancer. This causes the cells to switch to glycolysis to maintain ATP synthesis. It was also shown that NO reduced the amounts of reactive oxygen compounds (ROC) via raising NADPH and glutathione levels [30]. Additionally, patients with ovarian cancer had a worse survival rate when their transporter of glucose 1 (GLUT1) expression was elevated in advanced-stage OCa, and this correlates with poor prognosis. [31]. According to a study, inhibiting GLUT1 expression prevents ovarian cancer cells from growing both independently and dependently on anchorage and from producing glycolysis under stress [32]. Glycolysis has been linked to the transcription factor forkhead boxed protein M1 (FOXM1). Hexokinase 2 (HK2), GLUT1, and FOXM1 were all elevated in EOC [33].

Extracellular miRNAs may affect systemic metabolic regulation and act as potential indicators for metabolic condition and illness detection in wider physiological settings [34]. Further research revealed that HK2 is essential for the development of ovarian cancer. In ovarian cancer, HK2 was overexpressed. High-grade and advanced-stage malignancies are linked to HK2 expression [35]. By focusing on aerobic glycolysis, upregulation of miR-603 has been shown to reduce the malignant potential of ovarian cancer cells. By performing as a tumor suppressor, the miR-603 focuses on HK2 to influence cellular metabolism and prevent cancer [36].

De novo methyltransferase 3A (DNMT3A) was overexpressed in ovarian cancer tissues. Since DNMT3A suppressed the level of expression of the microRNA or enhanced the aerobic glycolysis process, cell proliferation, migration, and invasion of ovarian cancer, its overexpression was linked to miR-603 [36,37].

It was discovered that overexpressing LDHA in ovarian cancer reverses the inhibitory impacts of miR-383 [38,39,40]. The expression of several glycolytic enzymes (GLUT1, HK2, and LDHA) and their contribution to the promotion of ovarian cancer were assessed in a study by Xintaropoulou et al. This study also demonstrated that ovarian cancer proliferation and the growth of cells were decreased when the glycolytic cycle was inhibited using several glycolytic inhibitors [41]. Thus, these results demonstrated that the glycolytic system plays a significant metabolic role in ovarian cancer survival and progression and that ovarian cancer treatment may involve blocking the glycolytic process [42].

#### 1.1.2. Tricarboxylic Acid Pathway

Tricarboxylic acid (TCA) is present in the core of energy metabolism and is involved in macromolecule synthesis and maintaining redox balance. The TCA cycle is initiated when pyruvate generated from glycolysis is oxidized into acetyl-CoA by pyruvate dehydrogenase complex. The TCA cycle was deregulated in ovarian cancer. Studies showed that cancer cells rely on glutamine as a fuel instead of using the pyruvate that is generated during glycolysis.

**Figure 2:**
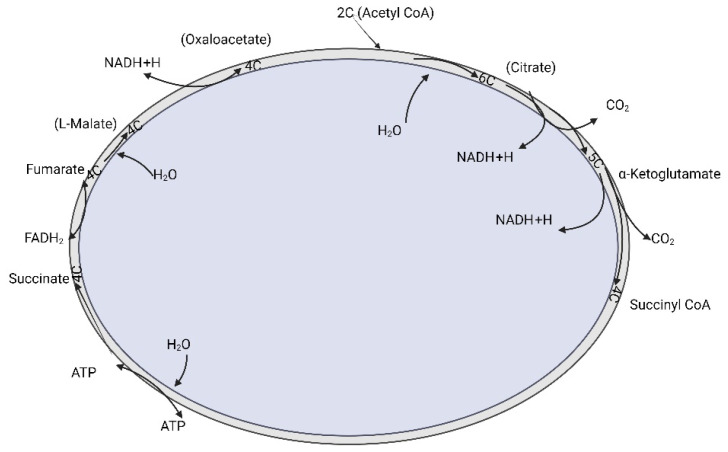
The tricarboxylic acid cycle (TCA cycle) is a cyclic reaction system consisting of a series of enzymatic reactions, starting with the formation of citric acid from acetyl coenzyme A and oxaloacetate, followed by four dehydrogenations, a horizontal phosphorylation of the substrate, and finally the production of two molecules of carbon dioxide and the reformation of oxaloacetate.

In addition, when there is impaired mitochondrial pyruvate transport, glutamine is used to regulate the TCA cycle and to meet the cells’ increased metabolic needs [43,44]. In ovarian cancer, invasiveness is correlated with glutamine dependence. Low-invasive ovarian cancer was glutamine-independent, whereas invasive ovarian cancer was dependent on glycine [45,46]. Fatty acid synthase (FASN) is an important enzyme that converts acetyl-CoA into saturated fatty acid. FASN was highly expressed in ovarian cancer, was associated with poor survival rate, and is upregulated in OCa and supports membrane biosynthesis, oncogenic signaling, and redox balance [47]. In different types of cancer, and especially in ovarian cancer, the genes encoding for the enzymes aconitase, isocitrate dehydrogenase (IDH), succinate dehydrogenase (SDH), and citrate synthase (CS) were deregulated, shown in Figure 2. Isoforms of IDH were identified to undergo missense mutation in different types of tumors, which include grade II/III gliomas and secondary glioblastomas (GBM), chondrosarcomas, and acute myeloid leukemia [48]. In ovarian cancer, wild-type IDH1 was unregulated in TCA cycle metabolism. [49]. Dahl et al. identified that HGSOC utilized glucose from TCA preferentially rather than from aerobic glycolysis. They also reported that IDH1 was upregulated in ovarian cancer and was associated with reduced progression-free survival. Targeting IDH1 modifies the histone epigenetic landscape, and this was discovered to induce senescence [50]. Bcl2-like-10 (Bcl2l10) is a member of the Bcl-2 family of genes that plays a key role in mediating apoptosis [51]. Knocking down of Bcl2l10 was reported to deregulate the TCA cycle as some of the components of the TCA cycle acted as a downstream target of Bcl2l10. Knocking down Bcl2l10 downregulated IDH1 and SDHD and led to the accumulation of oncometabolites, such as succinate and isocitrate, and therefore led to the promotion and progression of ovarian cancer [52]. Succinate dehydrogenase (SDH) is primarily involved in the catalytic conversion of succinate to fumarate by oxidation [53,54,55]. Chen et al. showed that silencing SDHB promoted cell proliferation, migration, and invasion, whereas SDHB overexpression suppressed cell proliferation and promoted apoptosis [56]. SDHB knockdown also led to altered glucose and glutamine utilization and caused mitochondrial dysfunction [57]. Citrate synthase (CS) was overexpressed in malignant ovarian tumors compared to benign tumors [58].

#### 1.1.3. Lipid Metabolic Pathway

Lipids are a heterogeneous group of organic molecules acting as an important source of energy, a key component of cell membranes, and they also participate in signaling processes. Fatty acid (Fas) forms the main building blocks for several lipid species that include phospholipids, sphingolipids, and triglycerides [59]. FAs maintain the cellular lipid homeostasis as well as regulate various biochemical processes.

Cancer cells are characterized by different alterations such as alterations in transport of FAs, lipid biogenesis, lipid storage, and β-oxidation. The most well-characterized Fas transporters include CD36 (fatty acid translocase), the members of solute carrier protein family 27 (SLC27), also known as fatty acid transport protein family (FATPs), and plasma membrane fatty acid-binding proteins (FABPpm) [60]. CD36 is a membrane glycoprotein expressed on the surface of cells. It was upregulated in different cancer types [61]. Ovarian cancer also exhibited FA uptake with the help of the CD36 transporter.

A study conducted by Ladanyi et al. showed that inhibiting CD36 showed the reduction in intracellular ROS levels in ovarian cancer [62]. FABP4, a transporter involved in direct transfer of lipids between adipocytes and ovarian cells, was upregulated in metastatic tumor sites. FABP4 was sufficient for diminishing the metastatic potential of HGSOC cells [63,64,65].

The de novo synthesized fatty acids are involved in various biological processes that include constructing and maintaining the cell membrane, forming molecules involved in cell signaling processes and biogenesis of energy-storing lipids [66].

**Figure 3:**
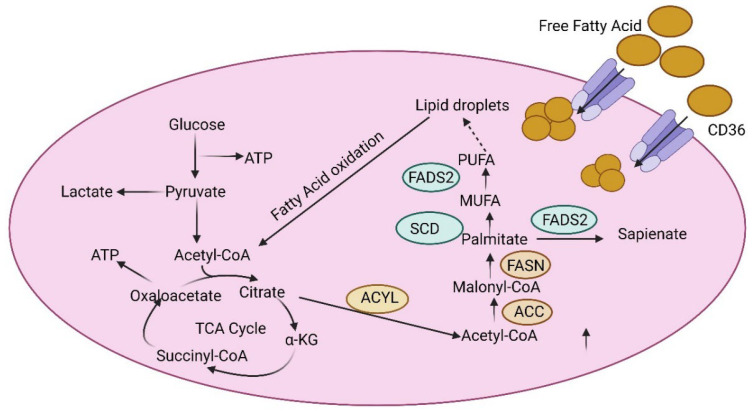
Fatty acid metabolism in the TME of ovarian cancer. Fatty acid-mediated lipid metabolism in TME is well controlled by cancer cells, adipocytes, and stromal cells with complex processes, leading to ovarian cancer metastasis and drug resistance. CAF, cancer-associated fibroblast; NK cell, natural killer cell; TAM, tumor-associated fibroblast; DC, dendritic cell; SREBP-1, sterol regulatory element binding protein 1; FABP, fatty acid binding protein; FASN, fatty acid synthase; SIK2, salt-inducible kinase 2; MCP-1, monocyte chemo attractant protein-1; TIMP-1, tissue inhibitor of metalloproteinase-1; and TGF-β1, transforming growth factor β1.

FASN and SCD1 are two critical enzymes in fatty acid production that are overexpressed in ovarian cancer [67]. The ectopic FASN stimulated growth in ovarian cancer cells, and the FASN levels and lipogenic activities were reported to affect cellular lipid composition. Veigel et al., found that benign ovarian tumors expressed more FASN than malignant ones. Although FASN is high in ovarian cancer, its prognosis seems to differ amongst histological subtypes [68,69]. Many studies reported that SCD1 played a role in maintaining the characteristics of cancer stem cells in ovarian cancer [70]. Inhibiting SCD1 in ovarian cancer promotes cell death by the processes of apoptosis and ferroptosis [71]. The increased SCD1/FADS2 levels tend to elevate the levels of PUFAs. SCD1/FADS2 maintained the ROS levels, as inhibiting them disrupted the cellular/mitochondrial redox balance by down-regulating lipid hydroperoxidase (GPX4) and the GSH/GSSG ratio shown in Figure 3 [72].

#### 1.1.4. Angiogenesis and VEGF Signaling Pathway

**Figure 4:**
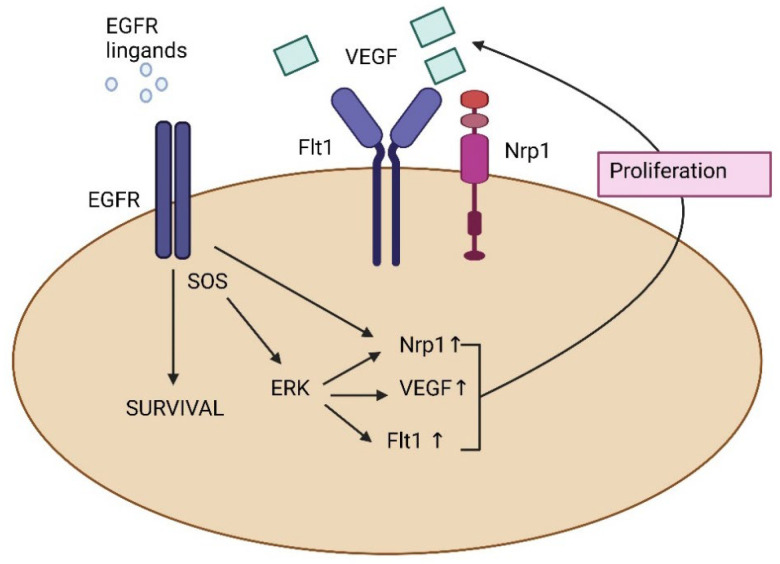
Activated Ras induces VEGFR, leading to cell-autonomous growth of skin tumor cells ► The autocrine function of VEGF in tumor growth is independent of angiogenesis ► VEGFR and EGFR signaling synergize to promote epidermal tumor growth ► A large fraction of human squamous cell carcinomas exhibits upregulation of VEGFR1.

Angiogenesis is the process of forming new blood vessels, which enables nutrients and oxygen to enter the surrounding tissues, thus promoting tumor cell proliferation, invasion, and metastasis [73,74,75,76]. Researchers have discovered that receptor tyrosine kinases (RTKs), VEGF and its receptor (VEGFR), and VEGFR2 or Flk-1/KDR RTK play key roles in pathological angiogenesis, particularly tumor neovascularization [77]. VGEF is a master regulator of angiogenesis, often upregulated via HIF-1α in hypoxic OC microenvironments. An immediate impact on tumor growth is observed (slowdown or stoppage) when the VEGF signaling pathway is blocked or inhibited [78]. This insight into the mechanism of angiogenesis led to the establishment of several treatment methods targeting the VEGF pathway shown in Figure 4 [79,80]. Current ovarian cancer clinical trials with bevacizumab show promising results (PFS) in two major first-line studies, ICON7 [80] and GOG 218 [81,82]. Other potential VEGF-targeting medicines, including soluble decoy VEGF receptors such as aflibercept (VEGF TRAP) [83] and VEGF kinase inhibitors such as sunitinib (SU11248, Sutent, Pfizer), have shown significant treatment benefit in EOC patients [84].

#### 1.1.5. ErbB Kinases Pathways

**Figure 5:**
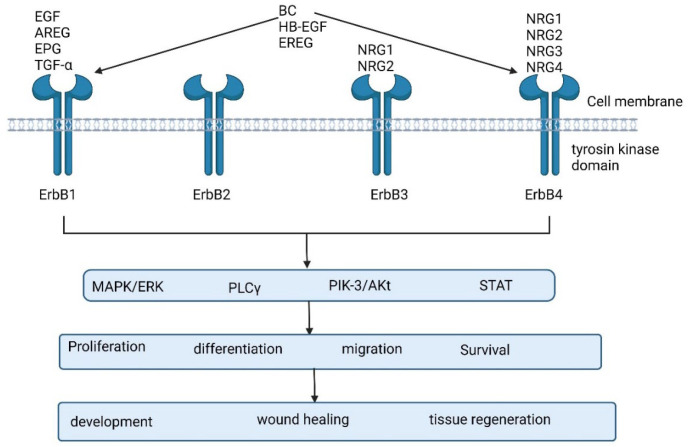
ErbB family kinase pathway.

The EGF family of RTKs, also known as ErbB or HER receptors, has been widely investigated in pharmacological research targeting human cancer. Numerous hypotheses have been suggested for HER2-mediated cell transformation through multiple mechanisms, such as EGFR and ErbB-3 interaction, which exhibit tyrosine phosphorylation and the activation of a cytoplasmic signaling pathway, while ErbB1 and ErbB2 homodimers transform fibroblasts using differential signaling [85,86]. According to the GOC study, trastuzumab had limited action in ovarian cancer [87]. A partial but long-lasting response was observed when combination therapy with trastuzumab–pertuzumab was used in a young woman with high-grade serous ovarian cancer (FIGO stage IV) [88]. Furthermore, Figure 5 shows many EGF-R targeting agents are currently in clinical trials [89,90,91], while some agents have shown exciting antitumor performance in CRC-based xenograft models and cell lines, such as cabozantinib, and are awaiting clinical trials [92,93,94] owing to PI3K-pathway-mediated tumor resistance through p38 MAPK activation and the following DNA repair [95]. Thus, targeting of EGFR, along with inhibition of p38 MAPK or DNA repair, may improve the efficacy of EGFR-mediated treatment in ovarian cancer.

### 1.2. Ovarian Cancer Microenvironment

The microenvironment of the tumor of ovarian cancer influences tumor growth, progression, metastasis, and therapy resistance. The TME is a complex network comprising immune cells, stromal cells, endothelial cells, and cancer-associated fibroblasts embedded in an extracellular matrix (ECM). All of these elements cooperate with ovarian cancer cells through physiological and biomechanical signals, producing a favorable environment for tumor formation and metastasis [96].

### 1.3. Natural Metabolites

Using diverse approaches, numerous research groups are trying to develop or find new treatment medicines against different types of cancer. Notably, during the preceding three decades, almost 50% of medications were licensed for use as therapies for ovarian cancer [97,98,99]. The organic substances that an organism produces naturally as a byproduct of its metabolic processes are known as secondary metabolites [100]. The useful components derived from plants, such as alkaloids, flavonoids, terpenoids, etc., may enhance multiple-drug sensitivity, thereby lowering the tumor and metastatic load of ovarian cancer significantly. Plant-derived products are currently being investigated as an adjuvant treatment to reduce the negative side effects of traditional anticancer treatment because of the unfavorable side effects connected with common anticancer drugs [101]. Our goal in writing this evaluation is to draw attention to the advantages of natural products, both alone and in combination for ovarian cancer [102,103,104,105,106,107,108,109,110,111,112,113]. This review examines the anticancer effects of several natural products as well as their molecular mechanisms in ovarian cancer therapy. An overview is given on the molecular mechanism of ovarian cancer, covering features such as autophagy, apoptosis, metastasis, proliferation, and sensitization, as well as the anticancer effects of many natural compounds.

### 1.4. Natural Metabolites with Their Mechanisms in Ovarian Cancer

Research into natural metabolites, derived from plants, has shown promising results in terms of their potential anticancer properties. These metabolites often have fewer side effects compared to conventional chemotherapy. Below are some natural metabolites and their mechanisms in ovarian cancer.

#### 1.4.1. Alkaloids

These are naturally occurring bioactive organic nitrogen-containing chemicals mostly found in plants. With complex ring structures and mostly alkaline characteristics, they have different clinical applications, such as analgesic, antibacterial, and anti-inflammatory actions, as well as relief from cough and asthma and anticancer capabilities [114].

##### Curcumin: (1E,6E)-1,7-Bis(4-hydroxy-3-methoxyphenyl)hepta-1,6-diene-3,5-dione)

**Figure 6:**
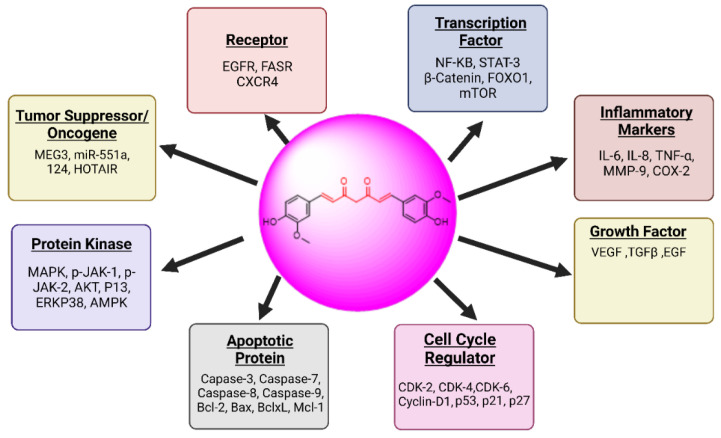
The main biological effects of curcumin on OC and their molecular targets.

It is obtained from *Curcuma longa* plant’s root, and curcumin interacts with several intracellular and extracellular molecules linked to different cancers. It may be able to halt the progression of cancer [115,116]. Curcumin has been shown to impact a number of cell signaling pathways, including those involved in the development of cancer: growth (HER-2, EGFR, or AP-1), the cycle of cells (cyclin D1 or cyclin E), swelling (NF-κB, TNF, IL-6, and IL-1, COX-2, or 5-LOX), cell death (caspase activation and reduction of anti-apoptotic peptides), survival (PI3K/AKT route), the growth of vessels (VEGF), invasion (MMP-9 or bonding molecules), or metastasis (CXCR-4), as shown in Figure 6. The majority of anticancer treatments available today are mono-targeted, and their usage has been restricted by their ineffectiveness, side effects, and high cost. Curcumin is a cheap, secure, and pleiotropic substance that could aid in the creation of multitargeted treatments [117,118].

Effect of Curcumin on Ovarian Cancer: Numerous common biological processes associated with gynecological cancer were identified by Gene Oncology (GO) enrichment analysis. The results of this analysis show that the biological processes associated with the control of the cell cycle and macromolecular metabolism are the ones that occur frequently in OCa [119]. Cells typically proceed through the G0/G1-S-G2-M cycle. Curcumin inhibits the disorderly growth of OCa cells by blocking their cell cycle at different stages [120,121]. When curcumin was applied to SKOV-3 cells, the number of cells in the G1/G0, S, and G2/M phases was reduced [122]. A study by Yu et al. [123] demonstrated that curcumin increased the production of caspase-3 and BAX via down-regulating the PI3K/AKT pathway, which stopped the progression of cells in the G2/M phase. Moreover, it has the ability to cooperatively trigger apoptosis and significantly lower the level of BCL-2. When triptolide and curcumin were combined, they effectively induced apoptosis by blocking the cellular cycle in S-phase and G2/M transition [124]. Nathan et al. added that curcumin causes cell death and cell cycle arrest in the G2/M phase by activating caspase-3 and PARP degradation, which raises the phosphorylation of p53. Previous research has indicated that numerous CDK/cyclins and CDK inhibitors (including p21 and p27) influence the cell’s G2/M phase [125]. A novel curcumin analog called HO3867 can control the production of the cyclin-dependent kinase 2 and cyclin, as well as p53, p21, and p27, which in turn activates caspase-8 and caspase-3 at the same time. It is responsible for arresting the G2/M phase of development in A2780 cells, which ultimately results in cell death [126]. Another substance, EF24, also shows tumor-suppressive properties in a range of malignant cancers. Through triggering of G2/M arrest and death, it significantly reduces the growth of CR cells. The prevention of pPTEN degradation, which is the protein encoded from the PTEN suppression gene, is likewise linked to EF24’s cytotoxic effect [100]. Overall, the aforementioned findings suggest that curcumin suppresses tumor cells’ uncontrolled growth by causing an arrest of cell cycle in the G2/M phase through a variety of mechanisms, including the control of proteins linked to the cell cycle and apoptosis.

These findings demonstrated that curcumin-induced autophagy prevents cancer cells from dying. Concurrently, Qu et al. [127] discovered that the novel monocarbonyl analog B19 of curcumin can cause OC cells to die through autophagy and apoptosis. Following combination therapy with curcumin and the autophagy inhibitor 3-MA, there was a significant increase in apoptosis. Consequently, it is anticipated that the combination for autophagy inhibitors or curcumin will overcome curcumin’s resistance to OC [128].

##### Quercetin

It is polyphenol phytochemicals commonly found in nuts, teas, veggies, herbs, and most foods that humans eat on a daily basis [129]. This polyphenol chemical has five hydroxyl groups that are present on the flavonol skeleton of quercetin, a pentalhydroxyflavonol, at positions 3, 30, 40, 5, and 7. Because of the substitution of its many functional groups, quercetin has a variety of biochemical and pharmacological properties [130]. As a result, quercetin influences many different molecules that play a role in the cell cycle. The PI3K-Akt/PKB pathway is implicated in multiple activities, including the regulation of cell survival, growth progression, and cell cycle, as well as carcinogenesis [131]. Several studies on quercetin have revealed its biological roles connected with its antioxidant [132], anti-inflammatory [133], immune-protective [134], anti-hypertensive [135], anti-carcinogenic [136], antidiabetic [137], anticancer [138], antiviral [139], antibacterial [140], and numerous properties [141].

**Figure 7:**
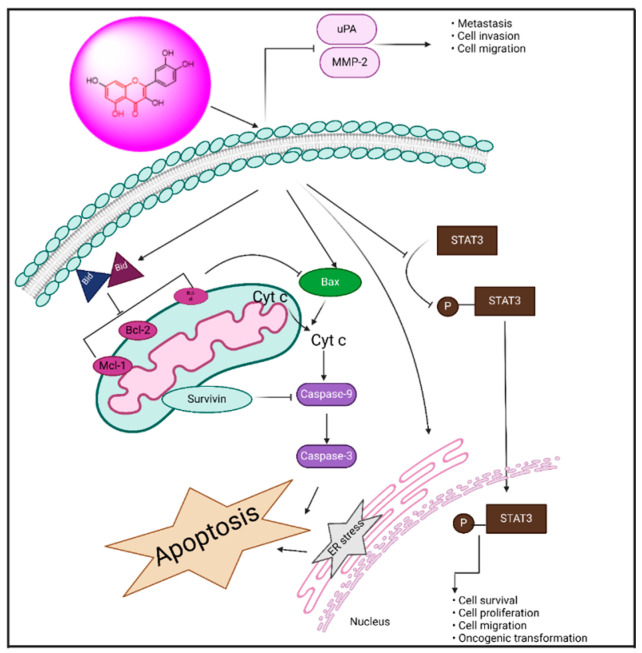
Schematic representation in targeting different signaling pathways using quercetin as a novel therapeutic strategy in the treatment of ovarian cancer.

Quercetin suppresses OCa proliferation by inhibiting growth and dose-dependent apoptosis [142]. After treatment of the OCa cells with 75 μM for 24 h, cell viability was reduced, and apoptosis was triggered [143]. Vascular endothelial-derived growth factors (VEGF) expression or receptor function contributes to tumor growth, invasion, and metastasis, and VEGF has become a new biological target related to OCa treatment [144]. Quercetin inhibits the growth of OVCAR-3 cells by modulating the VEGF [145]. Quercetin has additionally been shown to have effects on the pathway involving PI3K and Akt. A study found that quercetin regulates survival as well as the proliferation of cells via the PI3K/Akt/mTOR signaling pathway in OC [146]. It could down-regulate PI3K and Akt by binding to PI3K and inhibiting its enzyme activity as well as Akt concentrations in PA-1 cells. Furthermore, quercetin inhibits the phosphorylation of PI3K and Akt by lowering the production quantity of MMP-2/-9 within PA-1 cells, as shown in Figure 7 [121].

As a result, quercetin inactivates Akt, which reduces MEK and ERK protein levels. A study found that, via lowering survivin and PCNA protein expression levels, quercetin inhibited the survival or proliferation of PA-1 cells [147]. Furthermore, quercetin reduces the growth of metastatic OCa cells by enhancing mitochondrial-mediated apoptosis. Recently, nano-formulated quercetin effectively suppressed the proliferation in OCa cells via activating caspase-3, caspase-9, and Bax and lowering Mcl-1 or Bcl-2 levels in vitro and in vivo [148]. Moreover, quercetin causes apoptosis via increasing the expression of microbial RNA-145, which is engaged in the external death receptor-mediated and intrinsic mitochondrial pathway in the SKOV-3 or A2780 lines [149].

IRE1a-JNK signaling, which is implicated in the main endoplasmic reticulum stress pathway, is associated with quercetin-induced CHOP production and enhanced apoptosis in OCa cells through TRAIL. Quercetin-induced ER stress produced protective autophagy within the OCa cells via this p-STAT-3/Bcl-2 direction, which also played a role in the development of apoptosis [150].

##### Resveratrol: 5-(4-Hydroxystyryl) benzene-1,3-diol

Resveratrol is a naturally occurring polyphenolic compound produced by plants, including grapes, peanuts, and *Polygonum cuspi datum* [151]. Resveratrol has anti-inflammatory and anticancer effects that protect the heart, nerves, and kidneys [152]. Anti-proliferative and pro-apoptotic activity: The Warburg effect states that, under circumstances with sufficient oxygen, cancer cells are effective in the process of aerobic glycolysis [153]. Aerobic glycolysis generates a high number of metabolites and allows for abundant biosynthesis that may satisfy the quick and indefinite proliferation of tumor cells [154,155]. Resveratrol decreased hexosamine production in ovarian cancer cells, disrupted protein glycosylation by stimulating glycogen-synthase kinase 3β (GSK3β), or led to ER stress-induced death [25]. GSK3β is an enzyme that phosphorylates and deactivates glycogen synthase, an essential enzyme in the manufacture of glycogen [156]. Tino et al. [157] demonstrated that ovarian cancer cells’ growth and metabolism were more effectively inhibited by the combination of resveratrol or acetyl resveratrol. This growth restriction was brought about by a decrease in NF-κB protein or nuclear localization, which was in charge of VEGF (vascular endothelial growth factor) secretion [135]. Moreover, resveratrol reduced the levels of the extracellular signal-regulating enzyme (ERK) and phosphorylated AKT and GSK3β in ovarian cancer cells in a dose-dependent manner. This, in turn, repressed the activity of cyclin D1, which led to the promotion of cell cycle progression by cyclin-dependent kinases CDK4 or CDK6 [158].

**Figure 8:**
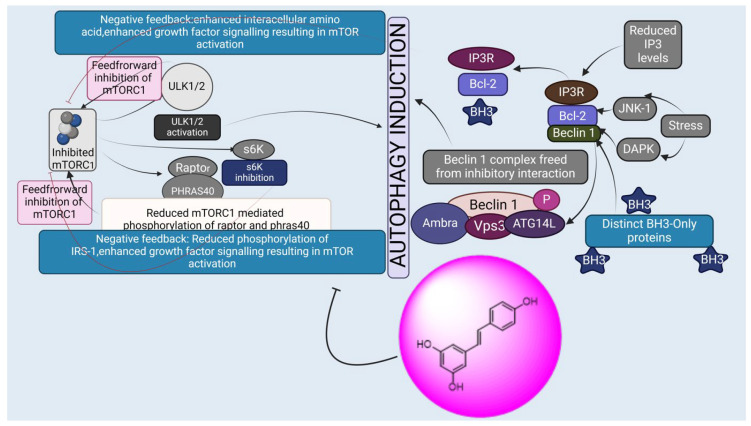
Mechanism of action of resveratrol in cancer management through modulating cell signaling pathways.

Induction of Autophagy: According to a recent study, resveratrol promoted autophagy and, as a result, apoptosis in cancerous ovarian cells by causing reactive oxygen species to be produced [159]. Additionally, it was discovered that resveratrol promoted degradation of microtubule-related protein one light chain 3 (LC3) I to LC3 II and increased the production of Atg5, a crucial component for the expansion of the autophagosome membranes, to induce autophagy (53). One particular marker protein indicating autophagic activity is LC3 II, which is found in the membrane of autophagosomes [160]. Through its interaction with different proteins, Beclin 1 controls the development and production of autophagosomes as well as modulates the location of autophagy-related proteins. Beclin 1 may also exert crosstalk between autophagy and apoptosis by interacting with the anti-apoptotic amino acids of the Bcl 2 family [161]. Zhong et al. [162] showed that resveratrol dramatically promoted growth arrest and apoptosis of ovarian cancer cells and improved autophagy by boosting the production of Beclin 1 or LC3 II through STAT 3 inactivation [140]. Moreover, resveratrol increased the expression of aplasia Ras homolog subunit I (ARHI), a tumor suppressor gene [163], and stopped ovarian cancer cells’ STAT 3 signaling pathway from functioning. Resveratrol then caused growth arrest, enhanced autophagy activity, and triggered cell death [164]. Concordantly, Ferraresi et al. [165] said that resveratrol inhibited the metastasis caused by IL 6 in ovarian cyst tumor cells by upregulating Beclin 1 or LC3 through upregulated ARHI or inactivated STAT 3 [142]. In the setting of inadequate nutrition, induced autophagy promoted cell survival when the pathway of mTOR was blocked by resveratrol, most likely resulting in a dormant condition, as shown in Figure 8 [166].

##### Berberine: 9,10-Dimethoxy-7,8,13,13a-tetradehydro-2′H-[1,3]dioxolo[4′,5′:2,3] berbin-7-ium

**Figure 9:**
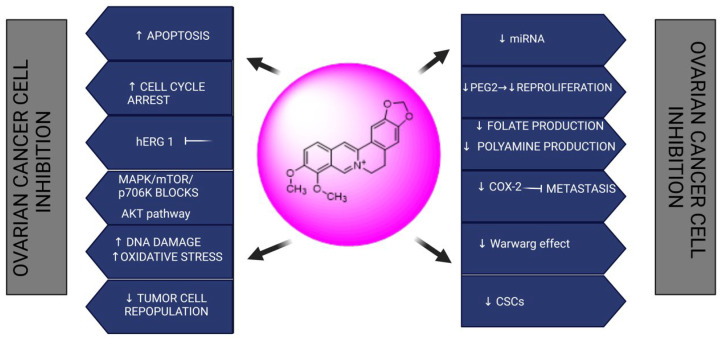
Chemotherapeutic activities of berberine in ovarian cancer.

Berberine, a yellow extract, can be extracted from a variety of plants including *Hydrastis canadensis* (goldenseal), *Coptis Chinesis* (Chinese goldthread), *Berberis aristata* (tree turmeric), *Berberis vulgaris* (barberry), and *Berberis darwinii* (Darwin barberry). It is a powerful autoxidizing agent having the potential to treat a variety of illnesses and hormonal imbalances. It shows numerous therapeutic activities including antimicrobial, anti-diabetic, anti-diarrheal, anti-hypertensive, anti-inflammatory, and hypolipidemic activities [167]. Evidence on berberine has also revealed that it can prevent the spread of ovarian carcinoma cells through several mechanisms, either alone or in conjugation with other chemotherapeutic agents, as revealed in Figure 9. Apoptosis Induction by Berberine: Potential therapeutic methods for malignant ovaries include medicines that target tumor cells and sensitize them to apoptotic signals. Berberine has been shown to promote apoptosis in a variety of cancers. One of the primary processes in the ovary is that berberine inhibits tumor development by increasing apoptosis, encouraging tumor differentiation, and blocking metastasis and invasion.

**Figure 10:**
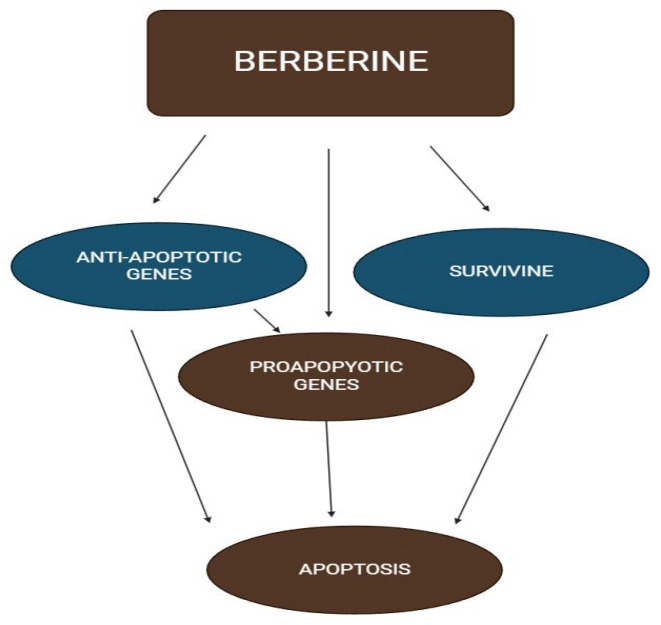
Schematic diagram of berberine showed the mechanism of apoptosis.

It has been demonstrated that a wide range of genetic, as well as epigenetic occurrences, can cause cancer cells to undergo apoptosis. In an investigational research, berberine significantly slowed the proliferation of cancerous cells by promoting apoptotic cell death in the ovary. Through suppressing the transcription of anti-apoptotic genes like BCL-2 and pro-survival protein and raising the expression of proapoptotic genes like BAX, berberine triggered apoptosis, as summarized in Figure 10. Additionally, cisplatin, along with berberine, had a cumulative influence on the growth of tumors [168].

A rise in the concentration of key proapoptotic proteins involved in apoptosis signaling pathways, including p53, Rb protein, serine/threonine kinase), caspase-8, Fas death receptor/Fas ligand, BH3 interacting-domain death agonist, and proapoptotic member of the Bcl. In contrast, it was found that after exposure to berberine, the levels of the apoptosis-inhibiting proteins, such as cIAP1, XIAP (an X-linked antagonist of apoptotic protein), Bcl X, and survival (an anti-apoptotic protein), decrease in Figure 10 [169].

##### Noscapine: (3S)-6,7-Dimethoxy-3-[(5R)-5,6,7,8-tetrahydro-4-methoxy-6-methyl-1,3-dioxolo[4,5-g]isoquinolin-5-yl]-1(3H)-isobenzofuranone

**Figure 11:**
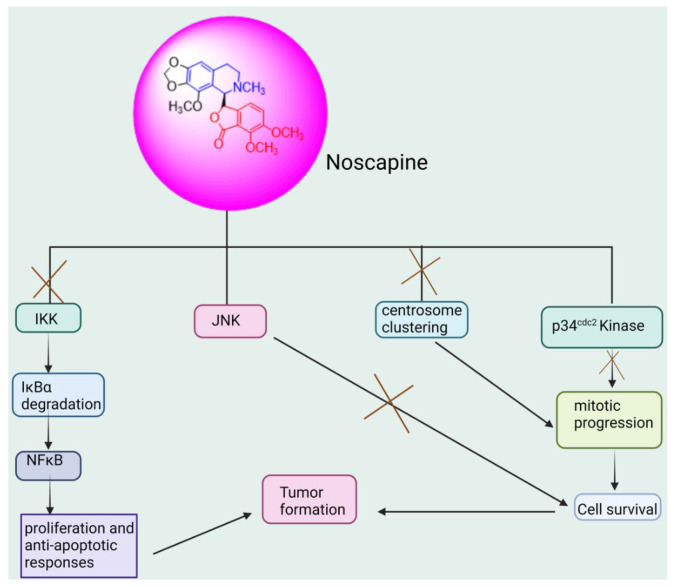
Anticancer pathway of noscapine targeting ovarian cancer.

Noscapine-induced necrosis in ovarian tumor cell lines was linked to the JNK pathway (shown in Figure 11), according to a recent study. According to this study, noscapine treatment of the cell lines raised c-Jun protein levels and caused JNK to phosphorylate it, which in turn affected the expression of apoptosis genes and proteins. Noscapine demonstrated potent anti-tumor efficacy, triggered apoptosis, caused mitotic arrest, and reduced microtubule dynamics similar to other microtubule medications [170,171,172]. The precise molecular processes behind the anti-microtubule agents-induced apoptosis and mitotic arrest, as well as the connection between these two processes, are still unknown. One of the main chemotherapy drugs for ovarian cancer, cisplatin, is well known for its extreme toxicity and ability to cause cancer cells to become resistant.

On the other hand, noscapine treatment increased drug-resistant ovarian cancer cells’ susceptibility to cisplatin [173]. When cisplatin and 2.5 µM noscapine were combined at varying dosages (0, 2, 4, and 8 µg/mL), ovarian cisplatin-resistant tumor cells proliferated less, and gene expression levels and anti-apoptotic protein levels dropped. And in comparison to utilizing either medication alone, there was an increase in the expression of the genes and pro-apoptotic proteins.

#### 1.4.2. Flavonoids

It is one of the three main secondary metabolites found in plants. Flavonoids are essential for human health and nutrition. Generally, the flavonoid components fall into seven categories: anthocyanidin, chalcone, flavone, flavanol, isoflavone, flavanone and flavonol. Numerous pharmacological actions are displayed by the substances, including anticancer effects, cardioprotective effects against cardiac diseases, antiviral activities, anti-inflammatory effects, and anti-aging effects [174]. The listed flavonoids have anticancer properties.

##### Apigenin: 5,7-Dihydroxy-2-(4-hydroxyphenyl)-4H-chromen-4-one

It has been reported that the dietary flavonoid apigenin has anti-tumor effects. Through the suppression of differentiation and DNA binding protein-1, apigenin prevents the growth and carcinogenesis of ovarian cancer cells in humans (Id1). Apigenin promoted transcription factors, which reduced the production of Id1 [175]. According to a different study on ovarian cancer, apigenin caused initial apoptosis in 24 h, while doxorubicin and α-mangostin caused late necrosis and apoptosis after 72 h. Furthermore, both α-mangostin or apigenin disrupted the cellular cycle in the phase of G2/M and significantly increased caspase-9 activity in apigenin-treated tumor cells at 24 h [176]. Another discovery indicates that apigenin was implicated in reducing the levels of Gli1 by inhibiting CK2α and inhibiting the self-renewal potential of SKOV3-derived SFCs [116].

Additionally, in both studied cell lines, apigenin increased the ratios of Bax/Bcl-2 and cleaved caspase-3/caspase-3 while decreasing mitochondrial transmembrane potential. The results demonstrate the molecular pathways underlying apigenin’s function in ovarian carcinoma cell death and resistance to cisplatinase [177].

**Figure 12:**
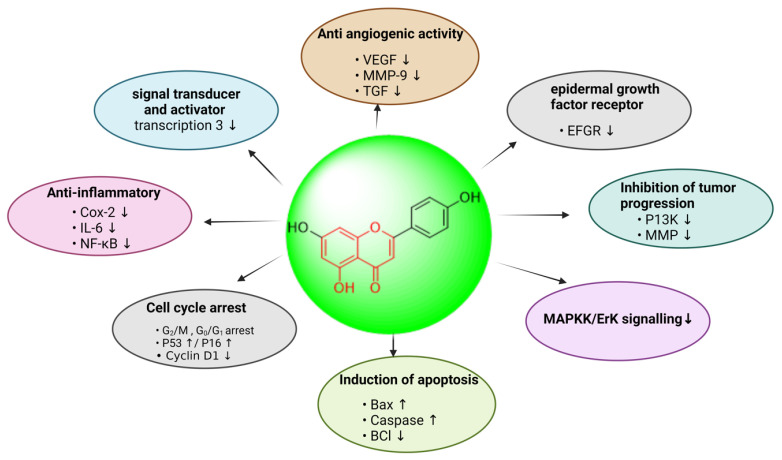
Apigenin’s role in cancer management through modulating cell signaling pathways.

According to a different ovarian cancer study, apigenin inhibited the growth of ovarian cancer by down-regulating the expression of ER-mediated PI3K/AKT/mTOR, indicating that it may be used as a therapeutic agent to treat ovarian cancer as shown in Figure 12 [178]. Apigenin significantly decreased the expression of matrix metallopeptidase 9, p-AKT, and p-p70S6K1 in malignant tissue as compared to the control group. Apigenin also downregulated Matrix metallopeptidase 9 through the AKT/p70S6K1 pathway. Furthermore, utilizing the orthotopic ovarian cancer model, it was revealed that the oral uptake of apigenin can limit tumor spread through the production of Matrix metallopeptidase 9 [179]. The complex cell nature of cancer is characterized by a number of complex molecular interactions and mechanisms.

##### Genistein: 5, 7-Dihydroxy-3-(4-hydroxyphenyl)-4H-chromen-4-one

Genistein has the ability to suppress a variety of cancer forms, including ovarian cancer. Throughout this study, we employed a model derived from the laying hen, a species well known for its high incidence of ovarian cancer that develops on its own. It was discovered that supplementing with genistein significantly decreased the number, size, and overall chance of developing cancer of the ovary. It has been demonstrated that genistein lowers oxidative stress markers, such as serum malondialdehyde, NF-κB, and Bcl-2 levels, in a study that looked into the molecular makeup of ovarian cancers. Conversely, it led to an upsurge in the amount of Nrf2, HO-1, and Bax protein expression in the ovary tissues. When genistein was consumed, there was a reduction in the overall amount of phosphorylation in mTOR, p70S6K1, and 4E-BP1, indicating a more restrained activity of the mTOR pathway. It is possible that genistein contributes to the chemotherapy used to treat ovarian cancer and highlights its impact on molecular pathways linked to the disease’s progression [180].

**Figure 13:**
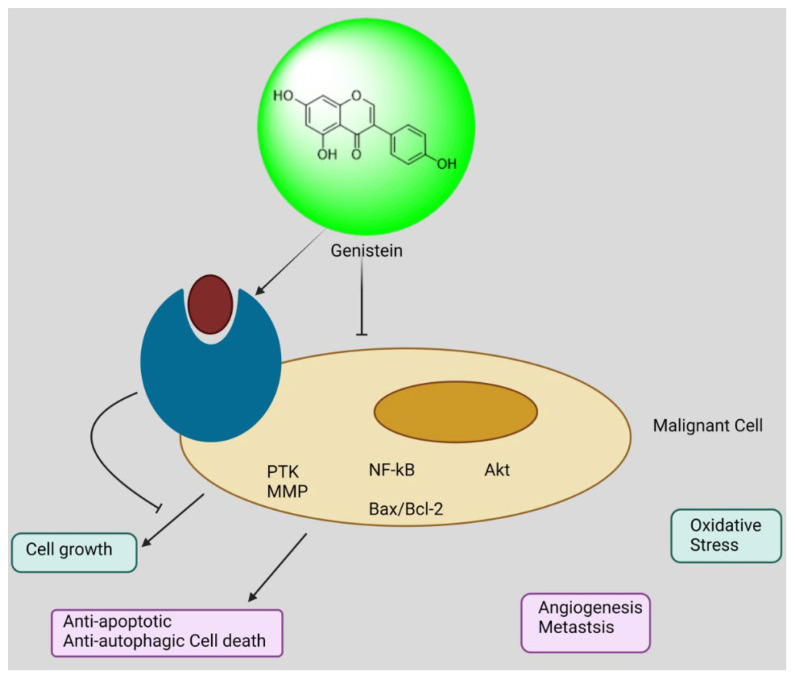
Pleiotropic effects of genistein on the inhibition of ovarian carcinogenesis.

Literature showed that genistein significantly improved the OVCAR-5 cells’ ability to proliferate and remain viable. Cellular mRNA and protein expression levels for the PCNA, cyclin D1, and CDK4 increased following genistein therapy, whereas levels of p21 and p27 decreased. Research has demonstrated that genistein can quicken OVCAR-5 ovarian cancer cells’ growth and G1-S transition [181]. The mechanism through which genistein affects the activity of the glycogen synthetase kinase-3 (GSK-3) route in the development of ovarian cancer is still unknown, although it reduces the risk of developing cancer. It has been demonstrated that genistein stops ovarian cancer cells’ growth. In the ovaries of mature laying hens with ovarian cancer diagnoses, research was conducted to ascertain the impact of genistein on biomarkers of inflammation and GSK-3 signaling pathways. Interleukin-6 (IL-6), interleukin eight (IL-8), tumor necrosis factor (TNF), and VEGF, or vascular endothelial growth factor, were among the inflammatory proteins whose blood levels were considerably higher in the control group when they were given the same amount of inflammatory protein as the experimental group. The outcomes demonstrated that these levels in the blood were dramatically reduced upon administration of genistein. Protein kinase B (p-AKT) and receptor substrate-1 (p-IRS-1) also increased after medication, although the effect on GSK-3 was the opposite. The amount of success that the treatment may have been directly impacted by the amount of the medication that is given. A decrease in pro-inflammatory biomarker levels and a suppression of GSK-3 expression in the ovaries of aged laying chickens demonstrated the anticancer effect of genistein. Research has shown that 7-difluromethoxyl-5,4′-di-n-octylgenistein (DFOG), a new synthetic genistein analogue that suppresses PI3K/AKT signaling (Figure 13) in vitro as well as in vivo, was more lethal in ovarian cancer cells when c-Myc was inhibited and linked to early-stage ovarian carcinoma [182].

##### Morin: 3,5,7-Trihydroxy-2-(2,4-dihydroxyphenyl)-4H-chromen-4-one

**Figure 14:**
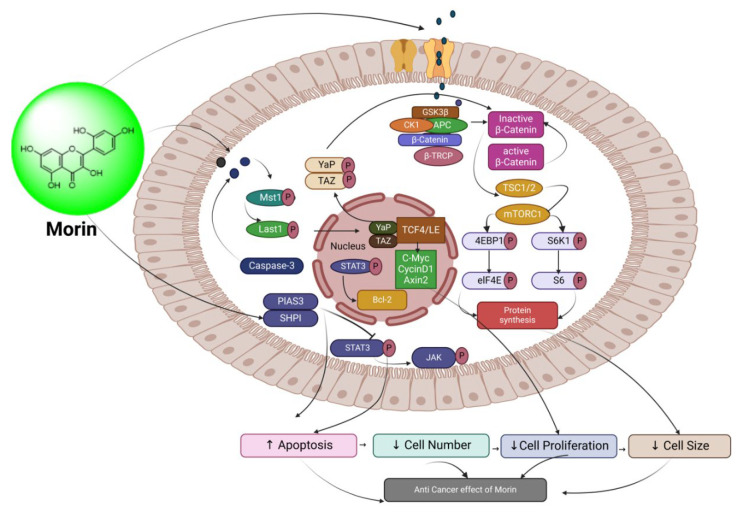
Schematic representation of signaling pathways in the ovarian cancer microenvironment impinging on the response of tumor cells to morin.

A polyphenol, morin, is a member of the flavanol family of flavonoids. Chemically speaking, it is 3,5,7,2′,4′-pentahydroxyflavone, which has been extracted as a yellow pigment from several plants in the Rosaceae, Fagaceae, and especially Moraceae families [183]. Among the numerous types of gynecological cancers, ovarian carcinoma has the greatest fatality rate due to a lack of viable treatments. Morin has been found in research to have a high anticancer impact and significantly reduced ovarian cancer proliferation and tumor size by suppressing the inflammatory response and regulating the NF-B signaling [184]. It was found to have anticancer properties targeting SK-OV-3 and TOV-21G ovarian tumor cells, inducing apoptosis while decreasing cell growth and viability. Similarly, another study reported the impact of morin, decitabine, and trichostatin on the migratory and adhesive potential, as well as the collection of G0/G1 phases A2780 cells, in SKOV-3 and A2780 ovarian carcinoma cell lines [185]. These findings established morin’s impact on upstream and downstream genes during EMT in SKOV3 and A2780 cells. Furthermore, investigations have revealed that morin may be a promising therapeutic drug against human cervical cancer via altering intrinsic and extrinsic signaling pathways, as shown in Figure 14 [186].

##### Wogonin: 5,7-Dihydroxy-8-methoxy-2-phenyl-4H-chromen-4-one

**Figure 15:**
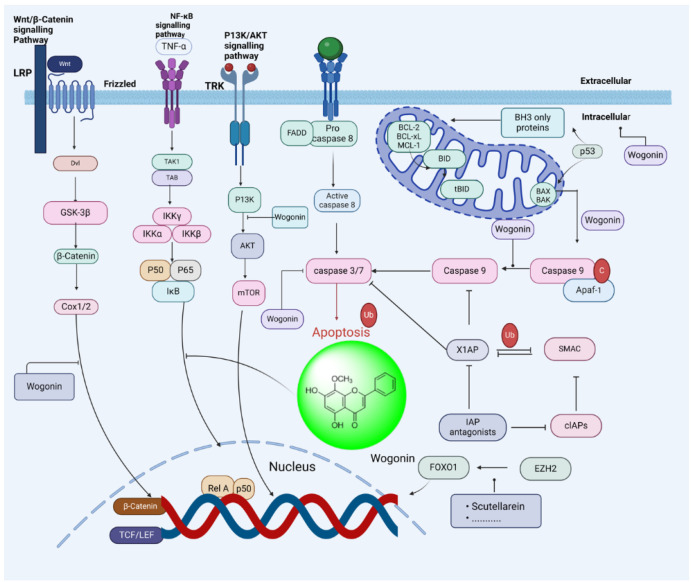
Anti-ovarian cancer mechanism of *Scutellaria baicalensis* and its natural compounds.

It is the primary bioactive component obtained from the plant’s roots of *Scutellaria baicalensis*. As a result of its broad range of pharmacological effects, including its anticancer, antiviral, antioxidant, antimicrobial, anxiolytic, and neuroprotective properties [187]. Wogonin primarily suppresses cell proliferation and encourages apoptosis in OC. The outcome demonstrated that wogonin therapy decreased the invasiveness and suppressed the proliferation of A2780 cells. Wogonin, in combination with MPP, a particular ER inhibitor, decreased the amount of expression of cyclin D1, CDK4, and CDK6 with a ratio of G0/G1, hence increasing the anticancer effects on A2780 cells [188]. It may reduce PGM, HK2, GLUT1, PDK, and LDHA and upregulate TIGAR and p53 in the transplanted cancer A2780 xenografts [189], as shown in Figure 15. Therefore, the above-mentioned study suggested that wogonin has good anticancer properties.

##### Baicalein: 5,6,7-Trihydroxy-2-phenyl-4H-chromen-4-one

**Figure 16:**
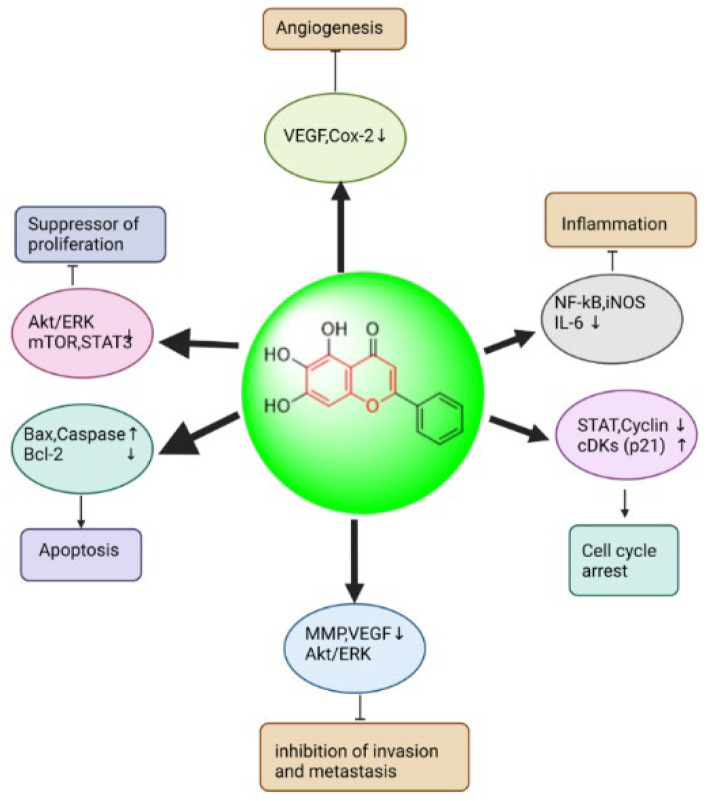
Anticancer effects of baicalein through the modulation of cell signaling pathways.

A phosphate chemical called baicalein (5,6,7-trihydroxy-2-phenyl-4H-chroMen-4-one) was isolated from the *Scutellaria baicalensis* (SB root). It is a trichophenolone composed of C-5, 6, and 7-bits. It has antimicrobial properties against cancer, viruses, bacteria, inflammation, and allergies. Its anticancer properties include inhibiting the processes that lead to cell proliferation and autophagic cell death [190]. Baicalein was found to have a weaker inhibitory impact on normal cells but to limit the viability and multiplication of cancer cells, potentially reducing the hazardous adverse effects of cancer therapy. Baicalein dramatically reduced the expression of HIF-1α and inhibited the production of pro-oncogenes, such as NF-κB, c-Myc, and others, as shown in Figure 16 [191].

Pan et al. studied the cancer-fighting effects of baicalein in A2780, SKOV3, and OVCAR cell lines. They found that baicalein lowered A2780 cell growth through the Akt/β-catenin signaling pathway and reduced the viability of all of these cell types. Baicalein not only prevented cell division but also brought on apoptosis. Through the intrinsic apoptosis pathway, baicalein caused apoptosis in A2780 cells. Paclitaxel boosted caspase-3 and PARP activity to promote apoptotic in human OC cells [192]. Wang et al. discovered that while treatment of HEY or A2780 cells with varying concentrations of baicalein only induced Beclin 1 or ERK-dependent autophagy in ovarian HEY cancer cells, treatment of HEY or A2780 cells with chloroquine or baicalein combined substantially decreased cell viability or increased PARP cleavage. Furthermore, they discovered that following baicalein therapy, phosphorylation of ERK, or Thr202/Thr204, and AKT increased. These findings imply that baicalein causes apoptosis and suppresses the growth of HEY cells [193]. According to Yan et al., baicalein dramatically slowed down OC cell invasion and decreased MMP-2 expression. Additionally, they suggested that baicalein prevented the activation of p38 and decreased the stimulation of NF-κB signaling molecules. Pyrrolidine dithiocarbamate (PDTC), in combination with baicalin, effectively decreased MMP-2 protein invasion and expression in OC cells via NF-κB signaling. In conclusion, baicalein’s anti-metastatic effects were demonstrated by its suppression of MMP-2 expression or OC cell invasion [194]. He et al. observed that baicalein had an intermediate inhibitory effect on the protein’s expression of vascular endothelium cell growth factor in their investigation of biologically active phenol compounds, which showed the highest suppression of OVCAR-3 and A2780/CP70 ovarian cancer cell proliferation [195].

#### 1.4.3. Terpenoid

With more than 50,000 known, terpenoids are crucial components of naturally occurring chemical substances. Strong antibacterial, anti-inflammatory, and anti-tumor properties are exhibited by natural terpenoids. Sesquiterpenoids, diterpenoids, monoterpenoids, and triterpenoids that have one or more double or triple-bonded carbon atoms are among them. Plants, microbes, marine life, and some insects all contain them in large quantities [196].

##### Tanshinone: 6,7,8,9-Tetrahydro-1,6,6-trimethylphenanthro[1,2-b]furan-10,11(3aH, 11aH)-dione

One of the most common cancers in humans, ovarian cancer is the leading cause of death from gynecological cancers [197]. The treatment plan for ovarian cancer is frequently limited, and the diagnosis occurs later than expected due to a lack of sensitive and precise early detection tools [198]. Thus, research on the treatment of cancer has focused on the creation of antiapoptotic TCM monomers [199]. It showed that tan IIA could cause apoptosis by weakening the PI3K/AKT/JNK signaling cascade. Caspases 3, 8, and 9 cleavage excitations significantly boosted apoptosis. According to Huang et al. investigation, it caused a cell cycle arrest at the G2/M phase, reduced Bcl-2, raised Bax, accelerated the death of SKOV3 cells, and reduced the viability and proliferation of cells [116]. It showed that it could directly upregulate miR-205 and subsequently down-regulate survivin to cause apoptosis in TOV-21G cells [177]. Chang et al. verified that by upregulating the DR5 acceptor via the ROS-JNK-CHOP signaling pathway, tan IIA increased tumor necrosis TRAIL-induced apoptosis [200]. Tan IIA increased the impact of TRAIL by reducing survivin in ovarian cancer cells, as demonstrated by Lin et al. [201]. Tan IIA-induced downregulation of survivin is controlled by both the transcription process or proteasome degradation and necessitates p38 MAPK activation.

**Figure 17:**
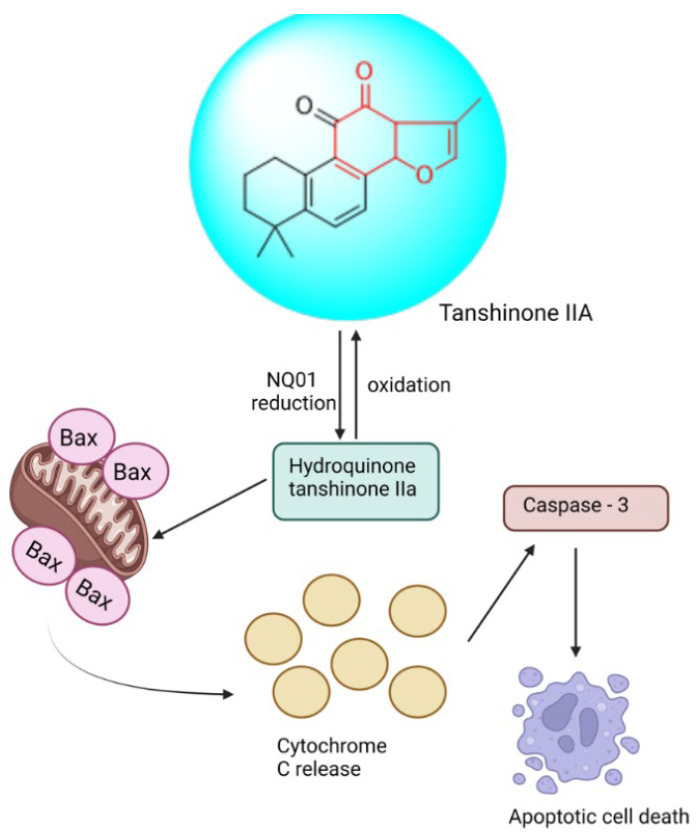
Anticancer pathway of Tan II.

Tan IIA has been shown by Jiao et al. to have a strong antiproliferative impact on COC1/DDP cells by inducing apoptosis and down-regulating genes that are resistant to cisplatin [202]. The main cause of the apoptosis was the decrease in survivin, and the decrease in ERCC1 and LRP encouraged reduced cisplatin resistance. (Its anticancer pathway is shown in Figure 17.)

##### Curcuma Oil: (1E,6E)-1,7-Bis(4-hydroxy-3-methoxyphenyl)hepta-1,6-diene-3,5-dione

**Figure 18:**
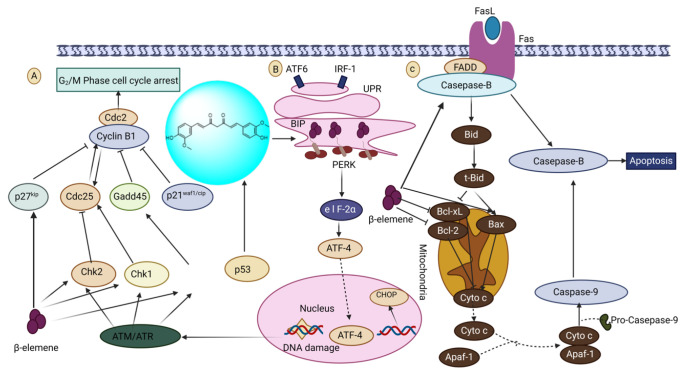
The anti-ovarian tumor effects of terpenoids in curcuma oil (curcumol and β-elemene) by the modulated expression of specific target molecules/pathways. (A) β-elemene-induced G2/M arrest in ovarian cancer cells via the ATM/Chk2/p53 pathway. (B) Curcumol promoted ovarian cancer cells’ apoptosis by the PERK-CHOP branch of the endoplasmic reticulum stress pathway. (C) β-elemene stimulated apoptosis in ovarian cancer cells via mitochondria- and caspase-dependent cell death pathways.

The primary ingredient and most bioavailable component in curcuma oil is curcumol. Curcumin was found to have a synergistic effect with niraparib by increasing its chemosensitivity, and it was also able to inhibit the growth, invasion, migration, and epithelial–mesenchymal transition of ovarian cancer cells via regulating the expression of PAX8 [203]. Curcumol’s lipid-soluble physicochemical makeup, however, restricts its possible routes of administration and therapeutic efficacy [204]. An extensive variety of human tumor cells, including ovarian cancer cells, can have their growth suppressed by β-elemene [205]. Moreover, β-elemene significantly reduced cancer cells in humans but had only a little impact on human ovarian cells, inhibiting ovarian cancer cells differently than normal ovarian cells. Additionally, they revealed that β-elemene dramatically increased the susceptibility of cisplatin-resistant ovarian carcinoma cells to cisplatin for the first time [206]. A few tests are available to clarify this sensitization mechanism. According to a study, β-elemene modifies the amount and activity of cell-cycle regulatory components to mediate cellular G2/M cycle arrest. The second experiment suggested that β-elemene function in an ovarian cyst tumor cell is co-mediated through triggering the arrest of cell cycles in the G2/M phase and causing apoptosis since cell stalled in the G2/M stage often proceed to apoptosis [207]. Additionally, it has been suggested that the process of sensitization of β-elemene involves apoptosis generated by mitochondria and the caspase-dependent cell death pathway. Surprisingly, β-elemene has a better ability to trigger apoptosis in this model system than cisplatin does [208]. Recent research has demonstrated that β-elemene’s utility is partially derived from decreased DNA repair activity, and the activation of apoptosis signaling pathways are shown in Figure 18 [209].

##### Oleanolic Acid: 3β-Hydroxyolean-12-en-28-oic acid

**Figure 19:**
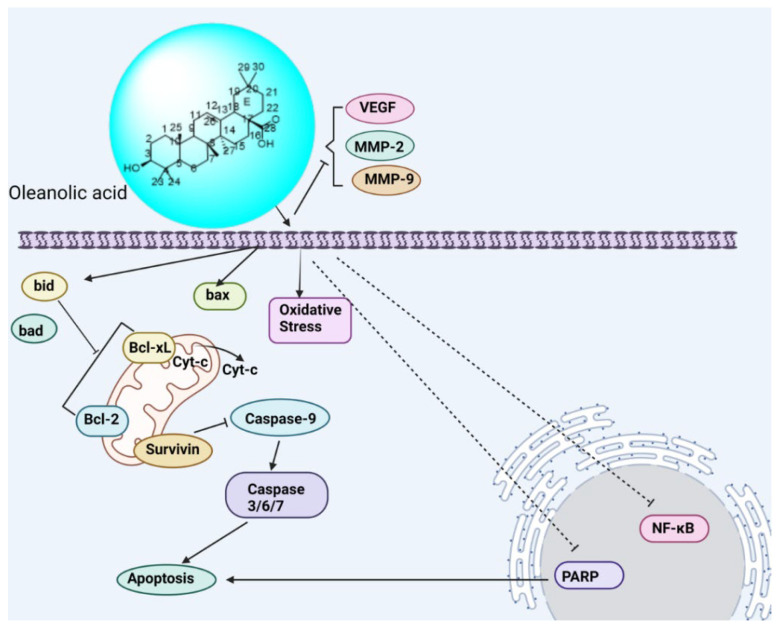
Schematic depiction of phytochemicals targeting distinct signaling pathways in ovarian cancer.

Oleanolic acid symbolizes triterpenes found in at least 1600 culinary and medicinal plants that have an oleanane skeleton [210]. One of the best places to get this chemical is in mistletoe herb. Five six-membered rings make up its carbon skeleton. As seen in the oleanane skeleton, it has a carboxyl group at position C-17, another hydroxyl group at position C-3, and, between the atoms of C-12 and C-13, a double bond. Because oleanolic acid has the three reactive functional groups indicated above, it can undergo a wide range of chemical changes that result in a wide range of derivatives [211]. Both by itself and in conjunction with cisplatin, oleanolic acid demonstrated apoptotic effects, such as an increase in reactive oxygen species (ROS), p38 protein mitogen-activated protein kinase (the MAPK pathway), and apoptotic signal-regulating kinase 1 (ASK1), and its suppression effect on human ovary cancer cell line A2780, A2780ZD0473R, and A2780cisR on signaling pathways such as ribosomal subunit S6 kinases (S6K), PI3K, mTOR, or Akt, and NF-κB (Figure 19). Because of these properties, oleanolic acid is more effective against cells resistant to platinum, preventing the start, spread, invasion, and vasculature of platinum-resistant ovarian cancer [210].

##### Artemisinin: 3,6,9-Trimethyloctahydro-3,12-epoxy[1,2]dioxepino[4,3-i]isochromen-10(3H)-one

**Figure 20:**
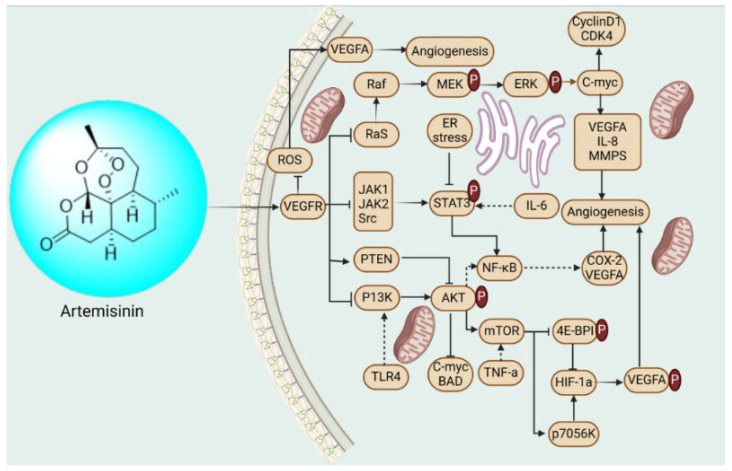
Schematic role of natural products in gynecological cancer angiogenesis.

*Artemisia caruifolia Buch*, a Chinese plant, is the source of the compound artemisinin [212]. Some studies have demonstrated that the expression of relevant genes and proteins was assessed using RT-qPCR and Western blot analysis with a concentration of 300 μM artemisinin. The findings revealed a significant down-regulation of ERa and VEGF expression (Figure 20) by artemisinin, thereby further confirming its potential antiangiogenic effect in cervical cancer cells. Moreover, artemisinin reduced telomerase activity, hTR and hTERT subunits, as well as the expression of HPV-39 virus E6 and E7 components, while increasing p53 expression. These results provide additional evidence supporting the reliance on p53 for artemisinin-induced apoptosis. Overall, these findings suggest that artemisinin exerts anti-proliferative and pro-apoptotic effects in HPV-39-infected ME180 cells. However, the lack of an in vivo experiment and the absence of a positive control group in this study preclude confirmation of artemisinin’s superiority [213,214].

The research studies mentioned in Table 1 show the natural metabolites for their toxicity profiles.

### 1.5. Clinical Trial Data on Natural Compounds for Cancer Therapy

1. Curcumin may be effective in preventing the development of colon cancer. Phase I trial is used to determine the dose of curcumin that can be tolerated to prevent colon cancer in healthy men and women [216].
2. Quercetin’s Potential for Treating and Preventing Chemotherapy-Induced Trial of Quercetin in the Treatment and Prevention of Chemotherapy-Induced Neuropathic Pain in Cancer Patients [217]. While quercetin is often considered safe, elevated dosages, especially those over 1 gramme day, have been linked to nephrotoxicity. This detrimental impact is mainly ascribed to oxidative stress and inflammation in renal tissues [218].
3. Resveratrol is purported to possess cancer preventive activity, especially for colon cancer, though its mechanisms of action are not well-defined. Clinical trial data are shown in Table 2.

### 1.6. Discussion

Natural metabolites are establishing themselves as potential modulators of sensing and signaling pathways in ovarian cancer, bringing up new therapeutic paths beyond traditional therapies. These bioactive chemicals affect oncogenic pathways such as PI3K/Akt/mTOR, NF-κB, and Wnt/β-catenin, which play important roles in tumor growth, immune evasion, and therapeutic resistance. They have the ability to impair the regulation of metabolism, and cellular communication systems make them suitable alternatives to traditional chemotherapy, improving efficacy for treatment whilst lowering toxicity.

Curcumin, quercetin, resveratrol, berberine, noscapine, apigenin, genistein, morin, and tanshinone are multifaceted compounds that regulate oxidative stress, inflammatory processes, and apoptotic networks. These naturally occurring metabolites impair tumor survival pathways, limit angiogenesis, and restore chemosensitivity in resistant ovarian cancer cells via regulating cellular signaling cascades. Moreover, their immunomodulatory activities enhance a more conducive tumour microenvironment, facilitating anticancer immune responses. Although preclinical studies highlight the synergistic potential of natural metabolites in ovarian cancer treatment, application is limited by challenges such as tumor heterogeneity, limited bioavailability, and drug resistance. Precision medicine offers a feasible framework to overcome these obstacles by tailoring therapies to specific molecular profiles. Tumor heterogeneity may be addressed by molecular subtyping and biomarker-directed treatment; limited bioavailability may be improved via nanoformulations and co-delivery systems, and resistance mechanisms can be targeted through dual-pathway blocking and metabolic reprogramming.

### 1.7. Conclusions

Ovarian cancer arises from metabolic reprogramming and dysregulated signaling pathways and leads to tumor growth, immunological evasion, and treatment resistance. Understanding the complicated interactions between metabolism and fundamental signaling networks might lead to novel treatments. Natural products’ ability to impact molecular targets makes them a promising treatment for ovarian cancer by disrupting metabolic and signaling relationships.

Utilising insights from metabolism, signaling pathways, and bioactive natural compounds such as curcumin, quercetin, resveratrol, berberine, noscapine, apigenin, genistein, morin, and tanshinone, future research may lead to the development of more effective and less toxic therapeutic strategies. These compounds influence key metabolic and signaling pathways, including PI3K/Akt/mTOR, NF-κB, and Wnt/β-catenin, suggesting their potential as adjuncts to conventional chemotherapy and their role in improving treatment outcomes in ovarian cancer management. This comprehensive strategy possesses the capacity to enhance patient outcomes and tackle the ongoing difficulties in ovarian cancer treatment.

## 2. Future Directions

In the future, the integration of multi-omics methods, including transcriptomics, proteomics, and metabolomics, will be crucial for mapping synergy networks, while machine learning algorithms may enable personalised predictions of optimum metabolite–drug combinations to improve therapy outcomes with minimal side effects.

## Figures and Tables

**Table 1:** The antitumor effect and mechanism action of natural compounds in ovarian cancer [215].

S. No.	Natural Product	Cell Line	Toxicity (mg/kg)
1.	Curcumin	SKOV-3	Immunotoxicity = 0.94 Cardiotoxicity = 0.59
2.	Quercetin	OVCAR-3	Neurotoxicity = 0.89 Immunotoxicity = 87
3.	Resveratrol	A2780	Carcinogenicity = 0.71 Neurotoxicity = 0.77
4.	Berberine	SKOV3	Respiratory toxicity = 0.76 Immunotoxicity = 0.99
5.	Noscapine	SKOV3	Carcinogenicity = 0.61 Nephrotoxicity = 0.71
6.	Apigenin	SKOV3/DDP	Respiratory toxicity = 0.75 Cytotoxicity = 0.87
7.	Genistein	OVCAR-5	Respiratory toxicity = 0.84
8.	Morin	SKOV-3 and A2780	Respiratory toxicity = 0.83
9.	Wogonin	A2780	Respiratory toxicity = 0.85 Cardiotoxicity = 0.82
10.	Baicalein	A2780, SKOV3, and OVCAR	Raspiratory toxicity = 0.83
11.	Tanshinone	SKOV3	Organ toxicity = 0.74
12.	Curcuma Oil	OVCAR-3	Respiratory toxicity = 0.8 Immunotoxicity = 0.90
13.	Oleanolic acid	A2780, A2780ZD0473R, and A2780cisR	Respiratory toxicity = 0.79
14.	Artemisinin	SKOV3	Immunotoxicity = 0.70

**Table 2:** Natural anticancer drugs under clinical trials.

S. No.	Compound Name	Title	Target	Agency	Phases of Clinical Trial	Compound ID
1.	Curcumin [219]	Curcumin for the chemoprevention of colorectal prevention	Cancer	University of Pennsylvania	Phase1	NCT00118989
2.	Curcumin and Paclitaxel [220]	Study investigating the efficacy of intravenous nanocurcumin (CUC-1^®^) in combination with paclitaxel for treating patients with advanced or metastatic breast cancer	Breast cancer	National Center of Oncology, Armenia	Phase 2	NCT03072992
3.	Quercetin [221]	Trial of Quercetin in the Treatment and Prevention of Chemotherapy-Induced Neuropathic Pain in Cancer Patients	Cancer	M.D. Anderson Cancer Center	Earlier phase 1	NCT02989129
4.	Resveratrol [222]	Resveratrol for Patients with Colon Cancer	Cancer	University of California, Irvine	Phase 1	NCT00256334
5.	Berberine [223]	Polycystic Ovary Syndrome (PCOS): Effect of Letrozole and Berberine	Cancer	Heilongjiang University of Chinese Medicine	Not applicable	NCT01116167
6.	Noscapine [224]	A Study of Noscapine HCl (CB3304) in Patients with Relapsed or Refractory Multiple Myeloma	Cancer	Cougar Biotechnology, Inc.	Phase 1	NCT00912899
7.	Apigenin [225]	Dietary Bioflavonoid Supplementation for the Prevention of Neoplasia Recurrence	Cancer	Technische Universität Dresden	Phase 2	NCT00609310
8.	Decitabine-Genistein Combination [226]	A Phase I/IIa Dose-Escalation Study of the Decitabine–Genistein Drug Combination in Advanced Solid Tumors and Non-Small Cell Lung Cancer (NSCLC)	Non-Small Cell Lung Cancer	Uman Pharma	Completed	NCT01628471
9.	Morin [227]	Contraceptive Pill and Hormonal Vaginal Ring in Women With Polycystic Ovary Syndrome	Cancer	University of Oulu	Phase 4	NCT01588873
10.	Tanshinone [228]	Tanshinone in Polycystic Ovary Syndrome	Cancer	Heilongjiang University of Chinese Medicine	Not applicable	NCT01452477

## Abbreviations

The following abbreviations are used in this manuscript:

GEPIA	Gene Expression Profiling Interactive Analysis
FOXM1	Forkhead box protein M1
HGSOC	High-grade serous ovarian carcinoma
p-IRS-1	Insulin receptor substrate-1
DFOG	Analogue 7-difluromethoxyl-5,4′-di-n-octylgenistein
MMP2	Myelin protein P2
